# Structure of the master regulator Rns reveals an inhibitor of enterotoxigenic *Escherichia coli* virulence regulons

**DOI:** 10.1038/s41598-021-95123-2

**Published:** 2021-08-02

**Authors:** Charles R. Midgett, Kacey Marie Talbot, Jessica L. Day, George P. Munson, F. Jon Kull

**Affiliations:** 1 Department of Chemistry, Dartmouth College, Hanover, NH USA; 2 Department of Microbiology and Immunology, Miller School of Medicine, University of Miami, Miami, FL USA; 3 Department of Biochemistry, Geisel School of Medicine at Dartmouth, Hanover, NH USA

**Keywords:** X-ray crystallography, Microbiology

## Abstract

Enteric infections caused by the gram-negative bacteria enterotoxigenic *Escherichia coli* (ETEC), *Vibrio cholerae*, *Shigella flexneri*, and *Salmonella enterica* are among the most common and affect billions of people each year. These bacteria control expression of virulence factors using a network of transcriptional regulators, some of which are modulated by small molecules as has been shown for ToxT, an AraC family member from *V. cholerae*. In ETEC the expression of many types of adhesive pili is dependent upon the AraC family member Rns. We present here the 3 Å crystal structure of Rns and show it closely resembles ToxT. Rns crystallized as a dimer via an interface similar to that observed in other dimeric AraC’s. Furthermore, the structure of Rns revealed the presence of a ligand, decanoic acid, that inhibits its activity in a manner similar to the fatty acid mediated inhibition observed for ToxT and the *S. enterica* homologue HilD. Together, these results support our hypothesis that fatty acids regulate virulence controlling AraC family members in a common manner across a number of enteric pathogens. Furthermore, for the first time this work identifies a small molecule capable of inhibiting the ETEC Rns regulon, providing a basis for development of therapeutics against this deadly human pathogen.

## Introduction

Diarrheal diseases are estimated to cause nearly 1.3 million deaths every year^1^. Enterotoxigenic *E. coli* (ETEC) is a leading cause of diarrhea, however there are various estimates of its prevalence. Older estimates suggested 280 million global infections with 380,000 deaths per year of children under five^2^, while newer estimates suggest only 75 million cases each year, killing up to 30,659 children under the age of five^3^. Despite this variation, ETEC is a leading cause of traveler’s diarrhea, a serious concern during military deployments, and remains a significant cause of childhood morbidity and mortality in low income nations^3–5^.

ETEC colonization is dependent on production of pili, which are sometimes referred to as colonization factors. ETEC pathogenicity is dependent on the attachment of these proteinaceous rods to the intestinal wall, and human challenge studies have shown that ETEC lacking adhesive pili are significantly attenuated^6–8^. Over 20 of these highly immunogenic colonization factors have been identified^9^, and while it has been suggested that vaccines incorporating them could be protective from ETEC infection^10,11^, to date such vaccines have not demonstrated protective immunity^10,12^. The lack of effective vaccines, coupled with the rise of antibiotic resistant strains that have associated treatment challenges and increased treatment cost^13^, highlights the importance of identifying new avenues for treatment.

While ETEC colonization factors are diverse, nearly half are regulated by the transcription factor Rns (also known as CfaD or CfaR), a member of the AraC family^14–22^. Rns activates pili loci expression by binding to a site immediately adjacent to the -35 hexamer, which may be accompanied by one or more additional sites further upstream^19,23^. Like most other AraC family members, Rns contains two helix-turn-helix DNA binding motifs in its carboxy terminal domain^21,24^, as well as an amino terminal domain that has been suggested to be involved in dimerization^25^ and ligand binding^21^. However, prior to this study ligand binding by Rns had not been experimentally demonstrated.

ToxT, an AraC family member from *V. cholerae* regulates transcription of genes encoding the two major virulence factors, the toxin-coregulated pilus (TCP) and cholera toxin (CT)^24,26^. Previous work in our laboratory found that unsaturated fatty acids (UFAs) inhibit ToxT activity when bound to a pocket in the N-terminal domain. This inhibition is due to disruption of ToxT’s ability to bind to promoter sites, as supported by the finding that the monounsaturated fatty acids oleic (C18) and palmitoelic (C16) acids inhibit ToxT DNA binding in vitro^27^. Because fatty acids are a component of bile, which is present in the GI tract of most organisms affected by *V. cholerae*, these compounds likely act as signals for virulence regulation^28^, and other virulence-controlling AraC family members could be inhibited by a similar mechanism^27^. A computational screen of AraC proteins identified several candidates for such regulation, including Rns.

In order to determine whether  Rns could be inhibited by UFA’s in a manner similar to ToxT, we solved the crystal structure of Rns. We observed two protein conformations, “open” and “closed” and show that Rns forms a dimer, consistent with the fact that it binds DNA as a dimer in a manner similar to other AraC proteins^29–31^. The open conformation contained a ligand bound in a groove between the N- and C-terminal domains, which we modeled as decanoic acid. Differential scanning fluorometry demonstrated that decanoic acid increased the melting temperature (*T*_m_) of Rns, supporting a model in which decanoic acid specifically binds to the protein. When Rns was crystallized with excess decanoic acid, only the closed conformation was observed. Furthermore, addition of exogenous decanoic acid abolished the expression of colonization factors by inhibiting Rns-dependent transcriptional regulation. These results support the hypothesis that Rns and ToxT utilize a common mechanism of binding fatty acid effector molecules to regulate virulence gene expression.

## Materials and methods

### Identification of candidate AraC proteins

**Figure 1:**
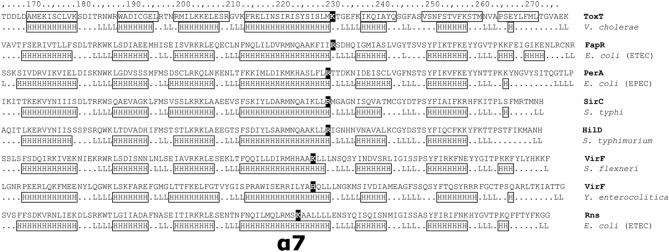
Structural alignment of the C-terminal domains of the top AraC family hits. ToxT is shown at the top with structurally determined α-helices shown in boxes. For the other proteins, H indicates predicted α-helices and L indicates predicted loop regions. The position of α7 is indicated and the positive residues are highlighted for each sequence. Predictions by the PROF algorithm using the server at http://predictprotein.org.

Fifty-eight proteins that had been previously characterized and identified as AraC family members were chosen for analysis^32^. Although these orthologs were linked to various functions (categories included: general metabolism, adaptive responses to nutrient sources, stress, and virulence), we analyzed all 58, regardless of functional class. As the prior study utilized sequence alignments for their analysis^32^, we used alignments of predicted secondary structure for our analysis. The following criteria were used to identify possible candidates: (1) The protein length was similar to that of ToxT. (2) The predicted secondary structure indicated a protein with an N-terminal β-strand rich domain and a C-terminal DNA binding domain comprised of seven α-helices (the canonical AraC DNA binding domain). (3) The presence of a positively charged amino acid at the C-terminal end of the α-helix predicted to be analogous to Lys230 in α7 of ToxT. (4) The presence of a positively charged amino acid in an appropriate position in the β-strand N-terminal domain. As β-strands are much more difficult to predict than α-helices, and as β-strand domains are inherently more variable, the exact location of this residue was less stringent than in rule #3. Using these rules, we identified four AraC virulence regulators out of the 58 that met all four primary criteria and for which comparison with an existing phylogenic tree^32^ showed they were among the closest relatives of ToxT. From members of the initial 58 candidates that failed our virtual screen based on rule #3, we identified three additional AraC proteins that are involved in virulence and have a positively charged residue near the end of the helix (Fig. 1, Table 1).

**Table 1:** List of potential ToxT like AraC proteins.

Protein	Organism	UniProt ID	Criteria met	Protein length	% Coverage/identity to ToxT
FapR	*E. coli*—ETEC	P23774	1–4	260	49/30
PerA	*E. coli*—EPEC	P43459	1–4	274	81/24
SirC	*S. typhi*	Q8Z4A6	1–4	295	34/30
HilD	*S.* *typhimurium*	P0CL08	1–4	309	40/24
VirF	*S. flexneri*	P0A2T1	1, 2, 4	262	84/28
VirF	*Y. enterocolitica*	P0C2V5	1, 2, 4	271	80/20
Rns	*E. coli*—ETEC	P16114	1, 2, 4	265	42/27

### Cloning of *rns* into an expression vector

The sequence encoding *rns* was optimized to remove rare codons and flanking sequences to insert into the plasmid pCDB24, a gift from Dr. Christopher Bahl (Institute for Protein Innovation), which contains a N-terminal 10xHis-SUMO tag (pCDB24 addgene.org), were added. The resulting construct was synthesized by IDT. The construct sequence was amplified using PCR and the resulting product was purified using the QIAquick PCR purification kit from Qiagen. The vector was digested with XhoI and purified using the QIAqucik PCR purification kit. The *rns* construct was cloned into the digested vector in frame with the SUMO tag, using the DNA Assembly Mix from NEB according to the manufacturer’s instructions, to create the 10xHis-SUMO-Rns (SMT-Rns) construct. The correct insertion of *rns* was verified by sequencing. The resulting plasmid was transformed into BL21 DE3 cells for unlabeled protein expression and into B834 DE3 cells for SeMet labeling.

### SeMet labeled protein expression

SeMet labeled protein was expressed using Selenomet media from Molecular Dimensions. The cultures were initiated from a frozen stock into Selenomet media supplemented with 50 μg/ml of l-methionine and 100 μg/ml carbenicillin. The culture was incubated at 37 °C overnight with shaking. The next morning the culture was centrifuged at 25 °C for 10 min at 3000×*g* to collect the cells. The cells were washed three times with water. After washing the cells were resuspended in a tenfold larger volume of Selenomet media supplemented with 50 μg/ml of seleno-l-methionine, and 50 μg/ml of carbenicillin. The culture was incubated at 37 °C till an OD600 of 1–2. The culture was induced by adding 500 μM IPTG and incubated at 18 °C overnight.

### Unlabeled Rns expression

SMT-Rns was expressed in modified TB media (Fisher Scientific). To start a frozen stock was used to inoculate a starter culture of 2 ml ZYP-0.8G media (ZYP media^33^ supplemented with 0.8% glucose) with 200 μg/ml carbenicillin, which was incubated overnight at 30 °C. The next morning of the starter was diluted 1:100 in TB containing 2 mM MgSO_4_ and 200 μg/ml of carbenicillin. This culture was incubated at 37 °C till an OD600 of 2–3. The culture was then diluted 1:10 in TB with 2 mM MgSO_4_ and 50 μg/ml carbenicillin in a 3 L baffled flask. This was again incubated at 37 °C till it reached an OD600 of 2–3. The culture was then induced with 500 μM IPTG and 5% glycerol was added. The culture was incubated at 18 °C overnight.

### Rns purification

SMT-Rns purification was performed as follows. The culture was pelleted at 3000×*g* for 25 min. The bacteria were resuspended in wash buffer (20 mM TRIS pH 8, 20 mM imidazole, 500 mM NaCl) supplemented with 500 μM EDTA, 500 μM PMSF, and a Roche protease inhibitor tablet. The culture was lysed by three passes through a French press. The lysate was clarified by ultracentrifugation at ~ 100,000×*g* for 45 min. The supernatant was filtered using a 0.45 μm filter, and 1 M MgCl_2_ was added to a final concentration of 1 mM.

The SMT-Rns was captured using a HisTrap column from GE Healthcare. The column was equilibrated with 10 CV of elution buffer (20 mM TRIS pH 8, 500 mM imidazole, 500 mM NaCl) followed by 10 CV of wash buffer. The supernatant was loaded onto the column at 2 ml/min. The column was washed with 10 CV of wash buffer followed by 9 CV of 9% elution buffer, and 2 CV of 20% elution buffer. SMT-RNS was eluted from the column with a 10 CV gradient of 20–100% elution buffer. Fractions were collected in tubes with EDTA for a final concentration of 100 μM.

Cleavage of the SMT-Rns was achieved using previously purified 6xHis-Ulp1-6xHis SUMO protease, a gift from Dr. Bahl (Institute for Protein Innovation). The relevant fractions from the His-Trap column were pooled and dialyzed against 2 l of (20 mM TRIS pH 8, 200 mM NaCl, 1 mM DTT, 500 μM EDTA) at 4 °C. After ~ 4 h the dialysis buffer was changed, and a 10 mg aliquot of the protease was added to the pooled fractions. Dialysis was continued overnight at 4 °C. The next morning a HisTrap column was equilibrated with 5 CV of dialysis buffer with 1 mM MgCl_2_ and 20 mM imidazole. Imidazole was added to the dialysate for a final concentration of 20 mM and MgCl_2_ was added for a final concentration of 1 mM. The dialysate was then applied to the column and the flow through was collected.

Final purification was carried using an HiTrap Sp ion exchange column (GE Healthcare). The column was equilibrated with 10 CV of Sp elution buffer (20 mM TRIS pH 8, 1 M NaCl, 250 μM EDTA) followed by 10 CV of Sp wash buffer (20 mM TRIS pH 8, 200 mM NaCl, 250 μM EDTA). Then the flow through from the cleavage step was loaded onto the Sp column. The column was washed with 10 CV of Sp wash buffer. Elution was performed using a gradient to 100% Sp elution buffer over 5 CV. The concentrations of the relevant fractions were determined, and DTT to a final concentration of 1 mM was added to the fractions.

### Crystallization and data collection

Initial crystal screening for the SeMet labeled Rns was done using 96 well block screens from either Qiagen or Hampton Research. Initial drops were setup either at 2 mg/ml or 0.6 mg/ml using a NT8 robot. The crystals were imaged using Rock Imager. The initial hits were then optimized. The best crystals of Rns were obtained in a base condition of 0.1 M (D/L) malic acid pH 7, with 6–10% PEG 3350. These conditions were then used in additive screens, Additive Screen (Hampton Research). The final crystallization condition for SeMet labeled Rns was 0.6 mg/ml protein added 1:1 to 0.1 M (D/L) malic acid pH 7, 6% PEG 3350, 0.01 M Betaine hydrochloride in sitting drops. The crystals were frozen using the well solution supplemented with 10% PEG 3350, and 40% glycerol as the cyro-protectant. Data was collected using the FMX beam line at NSLS-II. Two anomalous data sets were collected from the same crystal with 360º of rotation at a wavelength of 0.979184 Å.

Another set of screens were setup with native Rns at 0.6 mg/ml with 1 mM decanoic acid. The native Rns, decanoic acid mix was crystallized by adding the mixture in a 1:1 ratio to 0.1 M succinnic acid, 14% PEG 3350, 0.03 M glycyl-glycyl-gylcine in hanging drops. The crystals were frozen with the well solution supplemented with 30% ethylene glycol as the cryo-protectant. Diffraction data was collected at the AMX beam line at NSLS-II.

### Anomalous data processing and refinement

The anomalous data sets were processed using XDS^34^. The space group was determined to be P 2_1_ with a unit cell of 72.51, 49.97, 102.92, 90.00, 106.11, 90.00. The two datasets were combined in XSCALE^34^. The processed data was cut at 2.8 Å and used in a Hybrid Substructure Search followed by one round of AutoSol and one round of AutoBuild in PHENIX^35^. The model was refined using iterative rounds of automated refinement with Refine as implemented in PHENIX with NCS, secondary structure, as well as experimental phase restraints^35^, followed by manual model building using COOT^36^. Chimera and ChimeraX were used for model visualization^37,38^. Refinement statistics are listed in Table S1.

### Native data processing and refinement

The native data sets were processed using XDS^34^. The space group was P2_1_2_1_2_1_ with a unit cell of 48.24 95.15 133.09 90 90 90. This crystal had significant pseudo-translational symmetry with an off-origin peak 46% of the origin peak as determined by PHENIX^35^. The structure was solved using Phaser^39^ as implemented in PHENIX^35^ using the previously solved SeMet-Rns as a search model. After phaser there was visible density in the ligand binding pockets of both monomers. The density was still visible after a cycle of automated refinement in PHENIX^35^. Decanoic acid was added to the density using COOT^36^ and refinement was performed using iterative rounds of PHENIX Refine^35^ followed by manual model building in COOT^36^. Chimera and ChimeraX were used for model visualization^37,38^. Refinement statistics are listed in Table S1.

### Differential scanning fluorometry (DSF)

DSF was performed to assess the effect of fatty acids on Rns stability^40^. First stocks of fatty acids at 100× of final concentration were made by adding octanoic, decanoic, and palmitic acid to methanol. The decanoic acid was serially diluted by ½, in methanol, to obtain the concentrations for the dose response. 1 μl of the appropriate fatty acid was added to 99 μl of Rns, at a concentration of ~ 0.7 mg/ml, and incubated at room temperature for 1 h. Then 18 μl of the mixture was added to a PCR plate in triplicate. Sypro Orange dye (Life Technologies), diluted in buffer, was added to the PCR plate for a final concentration 5x, and a total reaction volume of 20 μl. For each condition a buffer only control was also performed in triplicate. The melting curves were generated using a StepOne + RT PCR machine (Life Technologies) with a gradient from 25 to 95 °C utilizing 1 °C steps. The normalized melt data was exported into STATA for analysis as described^41^.

### Plasmids

Plasmid pGPMRns-Myc is a derivative of pTags2 (Addgene) that expresses Rns-myc from *lacp. rns* was amplified from pGPMRns^42^ with primers 1519/1522. pTags2 vector backbone was amplified with primers 1414/1419. The two PCR products were Dpn1 digested then circularized with NEB HiFi. All plasmids used in this study are listed on Table S2 and oligo-nucleotides are listed on Table S3.

### Reporter strains

Plasmid pHKLac1 is a promoterless *lacZ* reporter integration plasmid with a *pir*-dependent origin of replication^43^. It carries *attP*_HK022_ for Int_HK022_-mediated integration into the chromosome of *E. coli* at *attB*_HK022_. The CS3 promoter was amplified from ETEC strain 1392/75-2a with primers 401/402. The CFA/I***,**** cexE and nlpA* promoters were amplified from ETEC strain H10407 with primers 38/40^44^, 394/395^45^, and 415/416^42^, respectively. The PCR products were digested with BamHI and EcoRI and then ligated into the same sites of pHKLac1 to construct pCS3Lac1 [CS3*p* (− 121 to + 352 relative to ORF)*::lacZ*], pCFAILac1 [CFA/I*p* (− 486 to + 343 relative to ORF)*::lacZ*], pCexELac1 [*cexEp* (− 549 to + 264 relative to ORF)::*lacZ*], and pNlpALac1 [*nlpAp*(-391 to + 58 relative to ORF*)::lacZ*]. Each reporter plasmid was then integrated into the chromosome of MC4100 *[F-araD139 ∆(argF-lac)U169 rpsL150* (StrR*) relA1 flhD5301 deoC1 ptsF25 rbsR]*^46^ as previously described^43^ resulting in strains GPM1072 (*attB*_HK022_::pCS3Lac1), GPM1061 (*attB*_HK022_::pCFAILac1), GPM1070 (*attB*_HK022_::pCexELac1) and GPM1080 (*attB*_HK022_::pNlpALac1). Colony PCR was used to verify that each strain possessed only a single plasmid integrant, as previously described^43^. Strains used in this study are listed in Table S4.

The λ Red recombinase system^47^ was used to generate a *rns* knockout in ETEC 1392/75-2a similarly to the *cfaD::kan* H10407 strain previously described^48^. Primers 1242/1150 were used to amplify a tetracycline resistance cassette targeting *rns* from pAH162. Electroporation of the cassette in to 1392/75-2a resulted in strain GPM3002 (*rns::tet*) which was confirmed via PCR and the loss of CexE and CS3 pilin expression.

### β-galactosidase assays

Lac reporter strains GPM1061, GPM1070, GPM1072, and GPM1080 were transformed with pGPMRns-Myc or vector pTags2 to determine the effects of decanoic acid on expression from Rns regulated promoters. All strains were grown aerobically at 37 °C to stationary phase in LB medium with 100 μg/ml ampicillin with or without decanoic acid in 0.4% vol/vol DMSO. β-galactosidase activity was assayed as previously described^49^.

### Analyses of protein expression

ETEC strains H10407 and 1392/75-2a were grown to stationary phase in CFA^50,51^ broth with or without decanoic acid in 0.4% vol/vol DMSO. Pilins were released from the outer membrane by incubating ETEC in 1/10 volume PBS at 65 °C for 20 min. Supernatants were clarified by centrifugation to remove insoluble material. Stationary phase whole cell lysates of ETEC or 5 μg pilin supernatants were subjected to SDS-PAGE then transferred to PVDF membranes or stained with 0.1% Coomassie R-250 in 40% ethanol, 10% acetic acid. PVDF membranes were blocked in TBS-Blotto (25 mM TrisCl pH 7.6, 150 mM NaCl, 5% wt/vol powdered nonfat milk). Antibodies against CexE homologs CexEα (H10407) and CexEε (1392/75-2a) were produced by immunization of rabbits (Proteintech Group, Inc.) with purified antigens and used at a dilution of 1:5000 in TBS-Blotto with 0.05% vol/vol Tween20^45,48^. The primary antibody against DnaK (AbCam ab69617) was used at a 1:10,000 dilution. HRP conjugated goat anti-rabbit (Santa Cruz Biotechnology sc-2030) and goat anti-mouse (Jackson ImmunoResearch 115-036-062) antibodies were used at 1:10,000 dilutions. Chemiluminescence and Coomassie staining was detected with an Odyssey FC Imaging System (LI-COR Biosciences).

## Results

### Identification of proteins potentially regulated by UFAs

Given the lack of overall sequence identity between ToxT and other AraC proteins we aligned secondary structure predictions and sequence alignments to identify eight AraC proteins that are known virulence regulators and that we predict may be regulated by UFAs (Fig. 1, Table 1). Many of these proteins did not contain a lysine in the area corresponding to Lys230 in ToxT, but rather an arginine, and in one case histidine. When these proteins were compared to an existing phylogenic tree of fifty-eight AraC family proteins^32^, all were found to be among the most closely related to ToxT. This supports our hypothesis that these AraC family members could share a common mechanism of being regulated by fatty acid ligands and served as a foundation for subsequent structural and functional analysis of these target proteins.

### Initial structure determination

**Figure 2:**
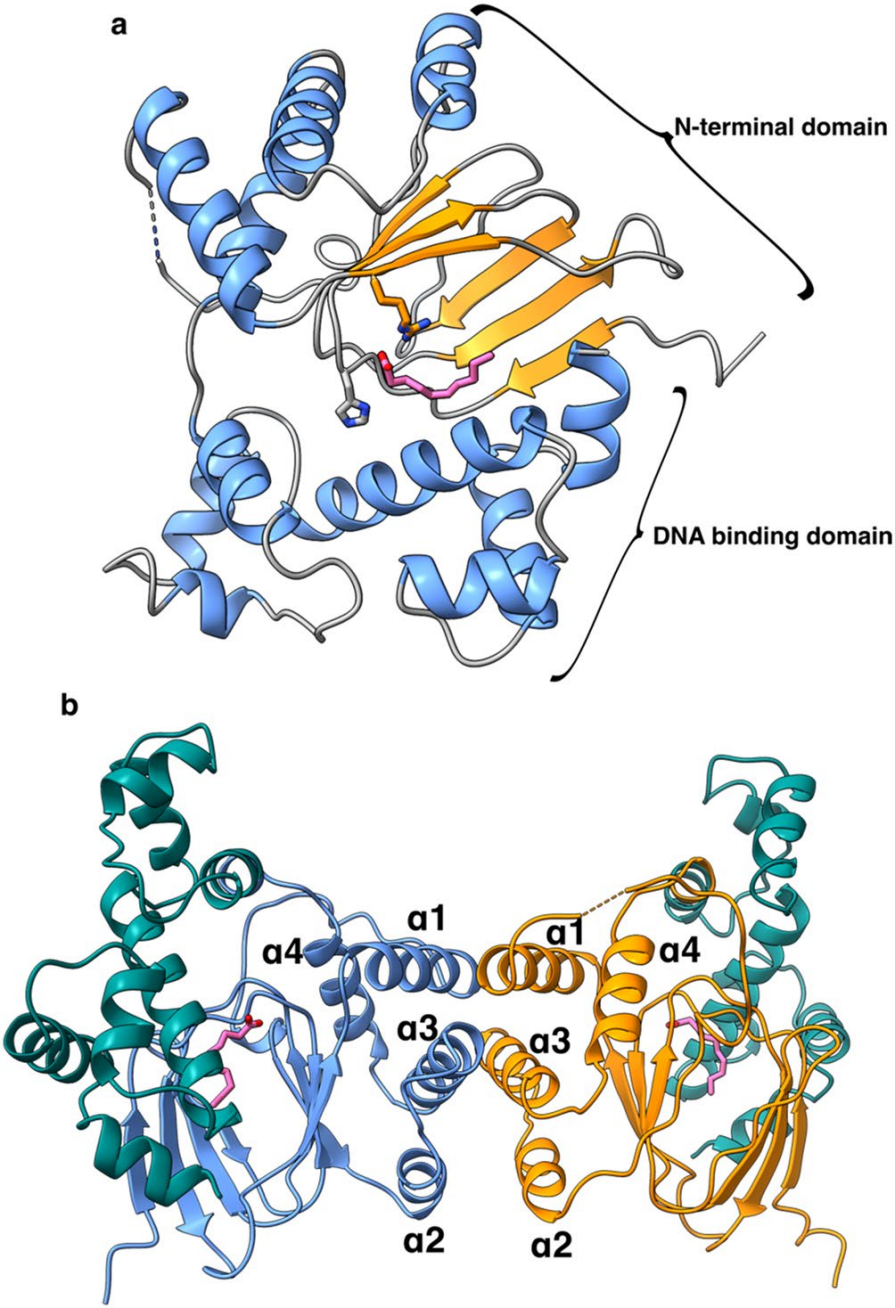
Structure of Rns and its potential dimerization interface. (**a**) Structure of SeMet-Rns showing decanoic acid modeled in the ligand binding pocket with α-helices in blue, β-sheets in orange, coils in gray, and the decanoic acid in pink. The N-terminal and DNA binding domains are labeled. (**b**) Proposed biological dimer with the N-terminal domains of the two monomers in orange or blue and both DNA binding domains in teal. The N-terminal α-helices are labeled. Images created with ChimeraX^38^.

We pursued several of these targets for biochemical and structural studies, and Rns was the first target that led to a crystal structure. Following crystallization and data collection, attempts at molecular replacement using ToxT and other AraC family member structures as phasing models were unsuccessful. Therefore, we used SeMet labeling coupled with single-wavelength anomalous dispersion (SAD) to experimentally determine phases and solve the structure. Rns shows the typical characteristics of two-domain AraC family proteins. The DNA binding domain (DBD) (residues 162–265) consists of seven alpha helices linked to the N-terminal domain by a 3 residue linker. The N-terminal domain (residues 1–158) has a cupin-like fold made up of 6 β-strands and 4 α-helices (Fig. 2a). One monomer in the asymmetric unit (ASU) was found to be in an open conformation in which the N- and C-terminal domains were separated by a groove containing electron density consistent with a bound ligand, which we modeled as decanoic acid.

### The Rns dimer interface

Rns formed a dimer across a crystallographic symmetry axis in a manner similar to other AraC proteins^30,31^. The interface consists of helices α1–α3 from the N-terminal domain of both monomers interacting in an antiparallel manner, burying approximately 274 Å^2^ of surface area (Fig. 2b). This is similar to the dimer interface of ExsA, an AraC family member from *Pseudomonas aeruginosa* involved in the transcription of the type three secretion system^31,52^. In Rns, the two DNA binding domains point almost 180° away from each other (Fig. 2b), which would allow the dimer to bind looped DNA in a manner similar to AraC^53^. Ongoing studies are focused on investigating the physiological relevance of Rns dimer formation.

### Rns contains a fatty acid ligand

**Figure 3:**
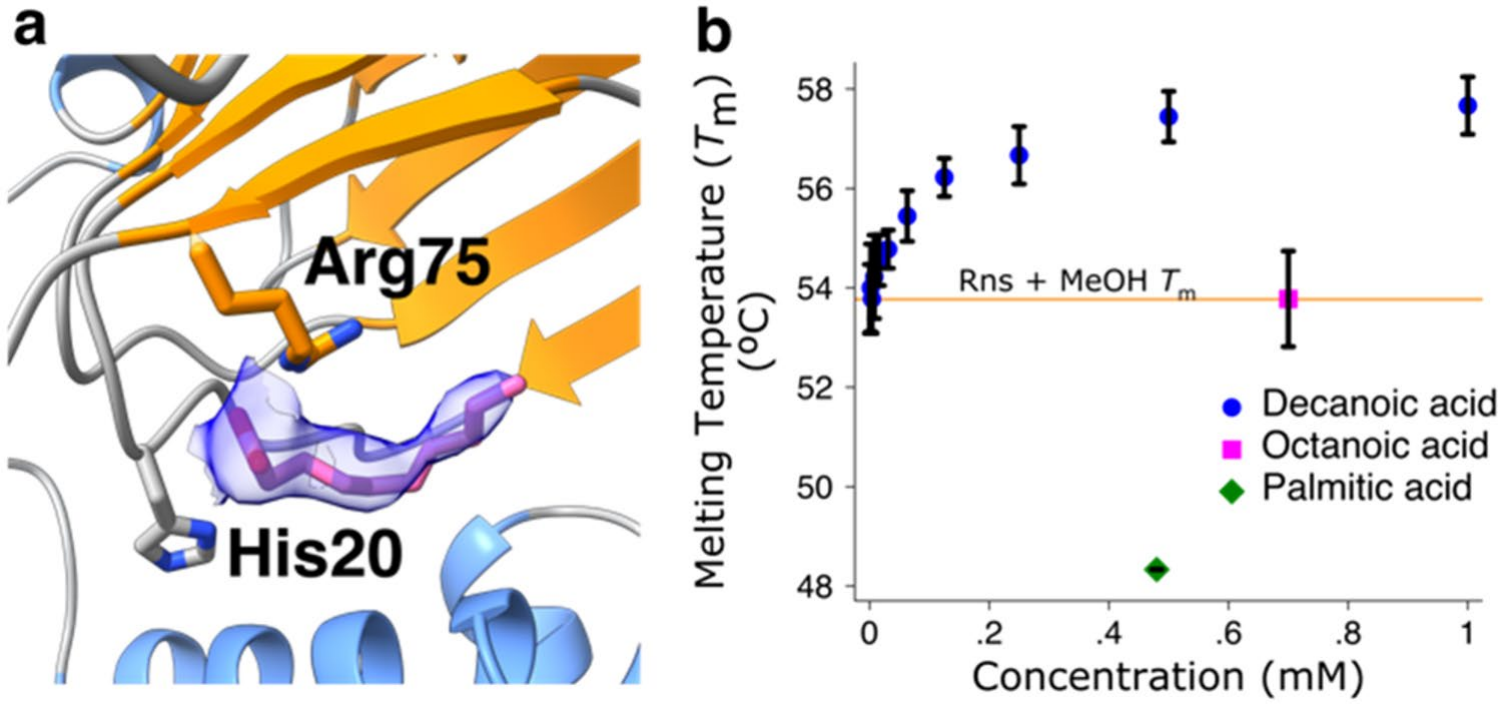
Rns binds decanoic acid. (**a**) Detail showing the electron density from an 2Fo-Fc omit map contoured to 1σ around the decanoic acid as a blue volume. Image created with ChimeraX^38^. (**b**) DSF showing an increase in the *T*_m_ of Rns in a dose dependent manner with decanoic acid. Octanoic acid does not affect the *T*_m_ of Rns and palmitic acid decreases the melting temperature of Rns. Data is shown as the mean ± SD, n = 3.

The crystal structure of the open conformation showed unexpected electron density consistent with the presence of a medium chain fatty acid. As we hypothesized Rns binds fatty acids, we initially modeled the density as eight-carbon octanoic acid. Following refinement, because the density was not completely filled by octanoic acid, we built in decanoic acid, which completely filled the electron density and further improved the refinement statistics (Fig. 3a). As no exogenous fatty acids were added to Rns during purification or crystallization, the presence of bound fatty acid suggested a physiological role; therefore we proceeded with analysis of the effect of fatty acids on Rns activity.

### Decanoic acid binds to and inhibits Rns activity

Differential scanning fluorometry (DSF) was used to determine if decanoic acid and other fatty acids could interact with Rns. DSF experiments were performed with decanoic acid (10 carbons), octanoic acid (8 carbons), and palmitic acid (16 carbons). While octanoic acid did not change the melting temperature (*T*_m_) of Rns, palmitic acid decreased its *T*_m_ and decanoic acid increased the *T*_m_ in a dose dependent manner up to a concentration of 1 mM (Fig. 3b). This is consistent with decanoic acid interacting specifically with Rns.

**Figure 4:**
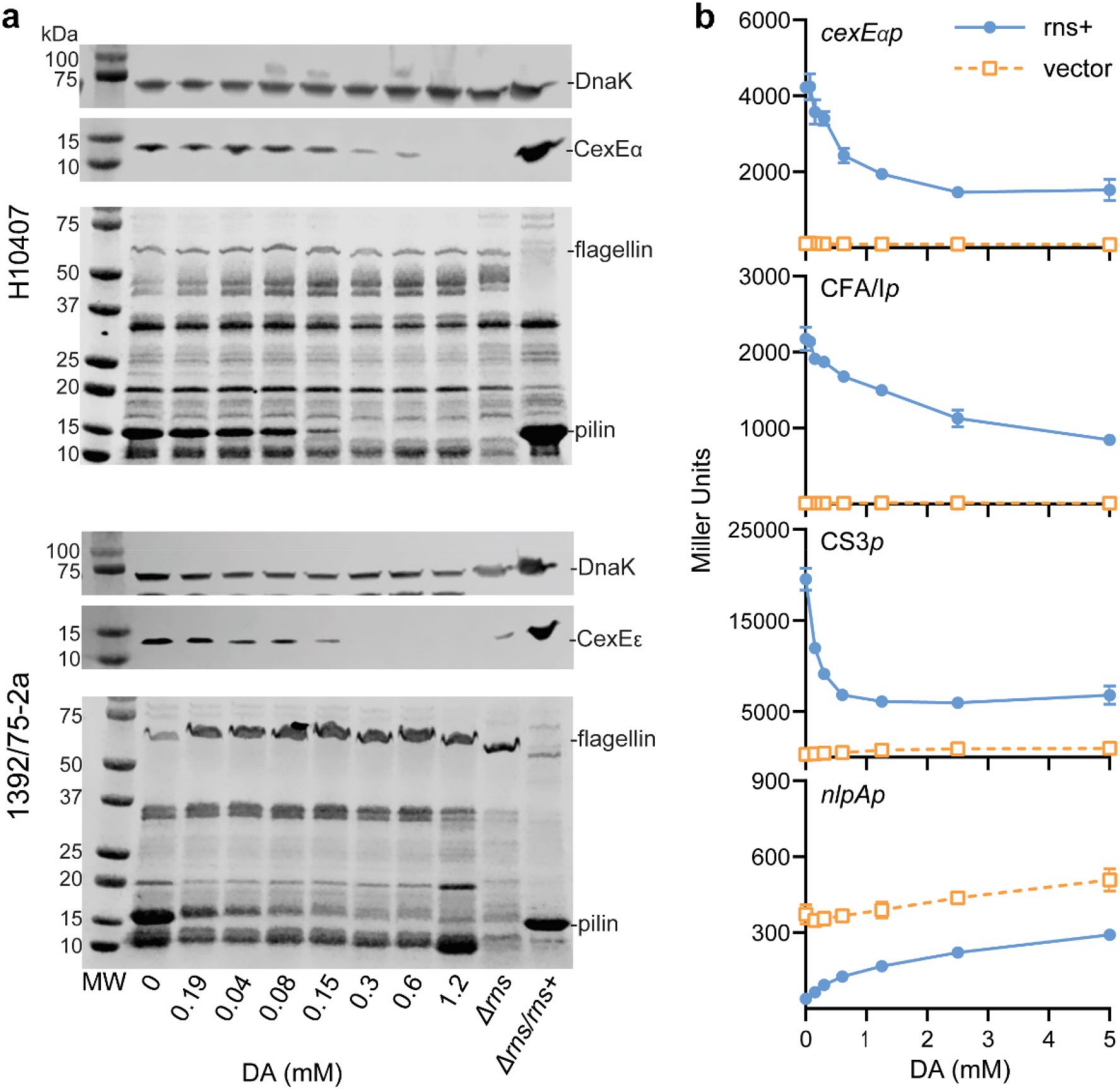
Decanoic acid inhibits Rns activity. (**a**) Excerpts of western blots of DnaK/CexE (same blot, probed sequentially for CexE then DnaK and cropped) and Coomassie stained pilin preparations from two ETEC strains (H10407 top and 1392/75-2a bottom). Expression of both CexE (12 kDa) and the major pilins (17 kDa) decreased with increasing concentrations of decanoic acid while DnaK (70 kDa) and flagellin (51 kDa) controls were unaffected. For full western blots see Fig. S1. (**b**) β-galactosidase assays showing decanoic acid repressed Rns activity at the *cexE*, CFA, and CS3 promoters and derepressed at the *nlpA* promoter. Data is given as the mean response ± SD, n = 3.

To determine the biological relevance of our in vitro studies we evaluated the effects of exogenous decanoic acid on the expression of Rns-dependent virulence factors with two different ETEC strains. Coomassie stained gels of outer membrane proteins released by heat shock revealed that decanoic acid inhibits the expression of both strains’ major pilins (Fig. 4a). This effect is specific to the Rns-dependent pilins because the expression of flagellin and other proteins of unknown identity was largely unaffected by the addition of decanoic acid. Likewise Rns-dependent expression of the outer membrane lipoproteins CexEα and CexEε^54,55^ was inhibited by decanoic acid in a dose dependent manner (Fig. 4a). The effects of decanoic acid on Rns-independent DnaK were negligible. This was expected because decanoic acid is not microbiocidal even at concentrations ca. 16-fold higher than the levels required to inhibit the expression of Rns-dependent virulence factors (Fig. S2). Thus, our in situ results reveal decanoic acid is a specific inhibitor of the Rns virulence regulon in pathogenic strains of ETEC.

Because we predict the effects of decanoic acid on Rns function should occur at the level of transcription, we employed Lac reporter strains to evaluate this hypothesis at Rns activated promoters^42,44,45^. As expected, the addition of decanoic acid inhibited Rns-dependent expression of β-galactosidase from *cexEp,* CFA*/Ip*, and CS3*p* in a dose dependent manner (Fig. 4b). Decanoic acid also relieved Rns-dependent repression of *nlpAp*, mirroring the effects of the activated systems (Fig. 4b). Although we also observed off target effects of decanoic acid, for example at *nlpAp* in the absence of Rns and the Rns-independent promoter *tibDBp* (Fig. 4b, Supplemental Fig. S3), the magnitude of these effects was less than when Rns was involved. As both repression and activation require Rns binding at sites near each promoter (Ref.^45^, and Munson, unpublished), these results suggest decanoic acid directly interferes with the ability of Rns to bind DNA. Thus, exogenous decanoic acid abolishes the expression of ETEC virulence factors by inhibiting the activity of the Rns transcription factor. This is the first evidence of a small molecule that specifically inhibits Rns mediated ETEC virulence and has significant implications for therapeutic applications.

### Structure with added decanoic acid

Given decanoic acid both stabilized and affected Rns function, we crystalized it in the presence of excess decanoic acid, ensuring saturation, to assess what effects this might have on its structure. Despite the diffraction data showing significant pseudo-translation, the structure was solved to 3.0 Å using molecular replacement with the apo SeMet-Rns structure as a search model. Analysis of the resulting electron density showed both monomers contained electron density consistent with a ligand, which we again modeled as decanoic acid.

**Figure 5:**
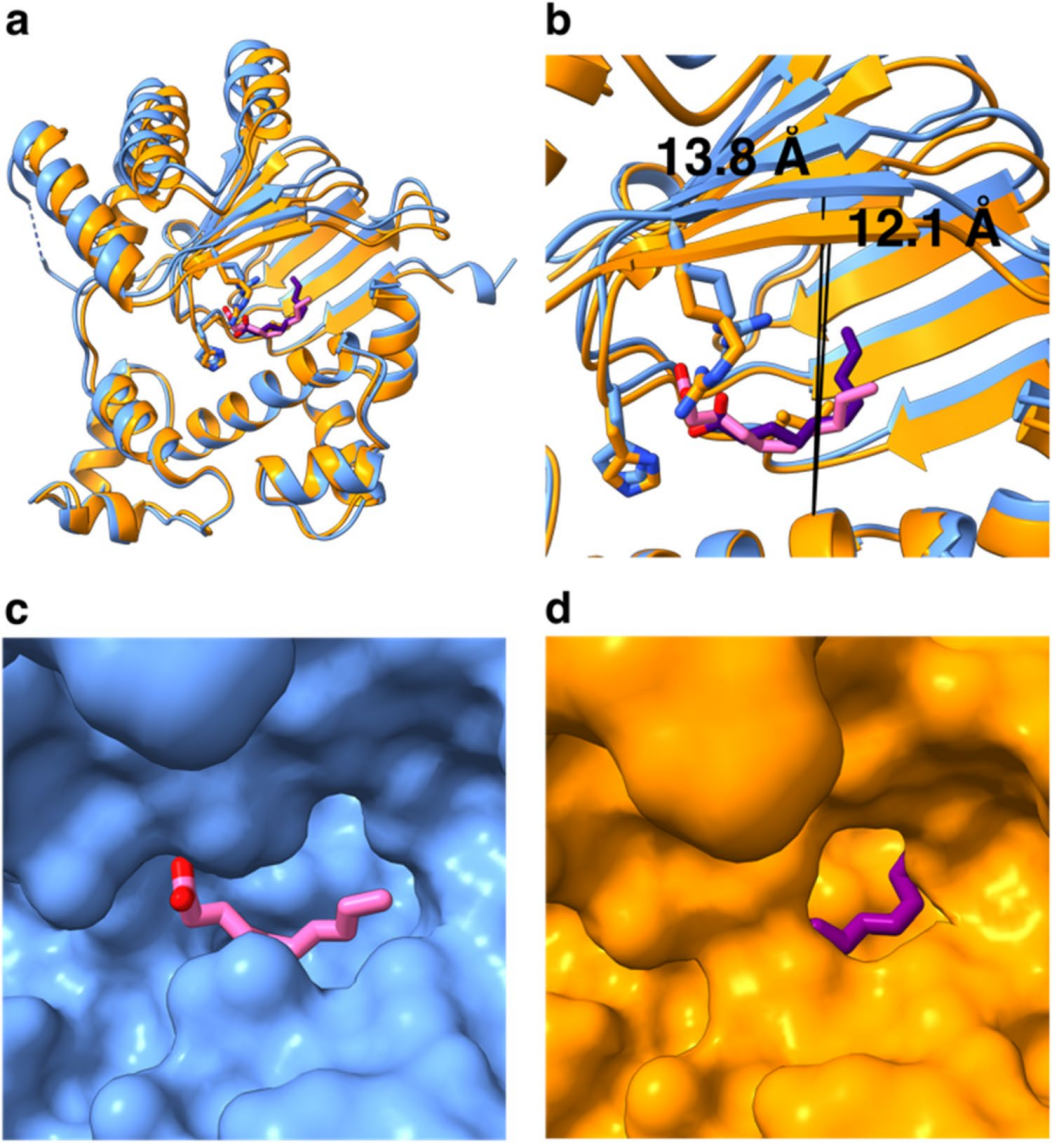
Comparison of the SeMet labeled structure in the open conformation and the native Rns structure in the closed conformation. (**a**) Superposition of the native (orange) and SeMet-Rns (blue) structures, the two domains were aligned using the long helix in the DNA binding domain (Phe205-Glu223), showing how the N-terminal domain is shifted away from the DBD in the SeMet structure. (**b**) Detail showing the β-sheet is shifted by 1.7 Å from the DBD in the SeMet structure (line to blue β-strand) compared to the native structure (line to orange β-strand). (**c**) Surface representation of the SeMet-Rns structure in blue with the decanoic acid in pink showing how the fatty acid is exposed to solvent. (**d**) Surface representation of the native-Rns in orange with the decanoic acid in purple showing how the ligand is enclosed in more of a pocket. Images created with ChimeraX^38^.

Overall, the SeMet and the unlabeled Rns structures are quite similar. In the unlabeled structure both monomers in the ASU were in the closed conformation. Therefore, we examined differences between the open monomer observed in the SeMet structure and a closed monomer of the unlabeled structure. The two conformations were aligned using the long helix in the DBD. This highlighted that helices making up the dimer interface and β-strands 2, 4, and 6 in the open conformation are shifted away from the long helix in the DBD by ~ 1.7 Å in comparison to the closed conformation (Fig. 5a,b). This shift results in a change from a pocket in the closed conformation to a groove in the open conformation (compare Fig. 5c to d).

### Arg75 and His20 in the structures make contact with the decanoic acid

**Figure 6:**
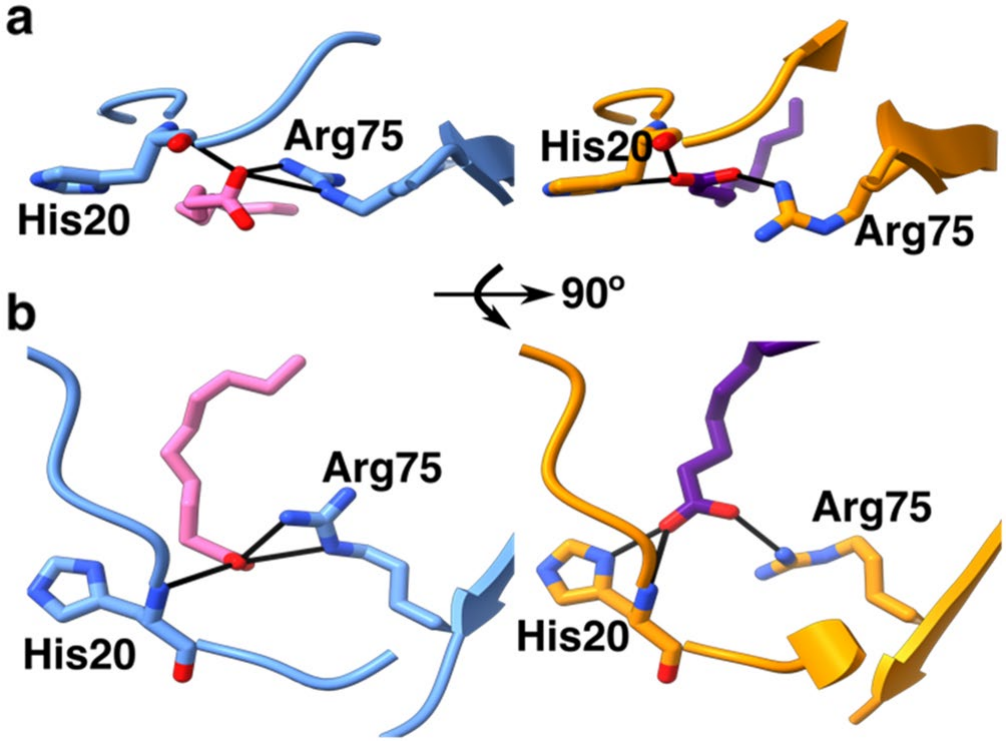
Detail showing the interactions between His20 and Arg75 to decanoic acid. (**a**) “front” view showing how the Arg75 in the SeMet-Rns, in blue, is above the decanoic acid (left panel) while in the native-Rns, in orange, Arg75 (right panel) is alongside the fatty acid. (**b**) “Top” view with the SeMet-Rns in blue (left panel) and native-Rns in orange (right panel). Images created with ChimeraX^38^.

In both the open and closed conformations, His20 and Arg75 interact with the carboxyl group of the decanoic acid (Fig. 6a,b). Notably, Lys216, which was the positively charged residue identified by our computational screen as potentially interacting with the fatty acid (Fig. 1), does not interact with the ligand. In the open structure, Arg75 is between the fatty acid and the rest of the N-terminal domain (Fig. 6a left panel), whereas in the closed structure, Arg75 is more alongside the ligand (Fig. 6a right panel). The position of the decanoic acid head group is in a different orientation in the open and closed conformations. In the open conformer, the head group is perpendicular to the plane of the imidazole group of His20, resulting in only one oxygen from the fatty acid making contacts with His20 and Arg75 (Fig. 6a,b left panel). In the closed conformation the fatty acid carboxyl group is turned 90° allowing both oxygens to make contacts with either His20 or Arg75 (Fig. 6a,b right panel). We speculate the position of the Arg75 drives the transition between open and closed conformations, and are currently exploring the role Arg75 and His20 play in mediating the structural response to decanoic acid binding.

### Comparison of Rns and ToxT binding pockets

**Figure 7:**
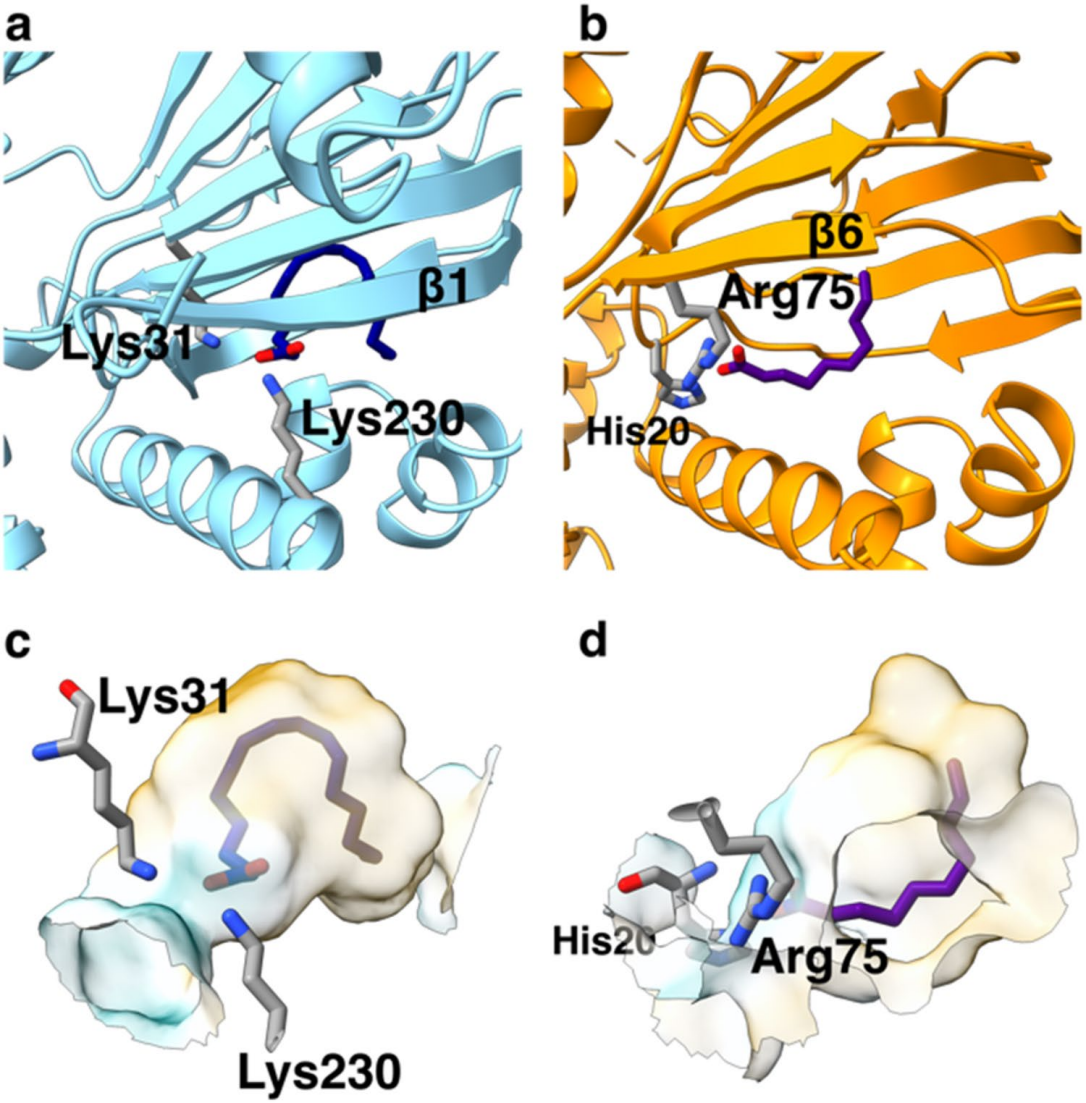
Comparison of the binding pockets of ToxT and Rns. (**a**) ToxT in blue with the palmitoleic acid in dark blue. (**b**) The native Rns in orange with the decanoic acid in purple were aligned in ChimeraX^38^ using the long helix in the DBD. The β-strand for each protein closest to the long helix in the DBD are labeled, as well as the residues which make contacts with the fatty acids. The binding pockets for (**c**) ToxT and (**d**) Rns are shown. The surfaces are colored according to lipophilicity calculated using ChimeraX^38^, with cyan for charged areas and orange for hydrophobic surfaces. Again, the residues interacting with fatty acids are labeled. Images created with ChimeraX^38^.

Because Rns and ToxT are the only full-length AraC structures solved that contain fatty acid ligands, we compared the binding modes of the two proteins. The proteins bind fatty acids of different lengths and degree of saturation: palmitoleate (16 carbon monounsaturated) binds to ToxT, while decanoate (10 carbon saturated) binds to Rns. We aligned ToxT and Rns using ToxT residues 211–231 (3GBG^27^) and the native Rns structure residues 205–223 with ChimeraX^38^. While both fatty acids are bound between the N-terminal domain and the DBD (Fig. 7), in ToxT the bound fatty acid projects more deeply into the N-terminal domain (compare Fig. 7a to b). In ToxT, residues from the N-terminal domain (Lys31) and the DBD (Lys230) interact with carboxyl group on the fatty acid (Fig. 7a), whereas in Rns the two residues that interact with the carboxyl group are in the N-terminal domain (Fig. 7b). The β1-strand in ToxT is absent in the Rns structure (compare Fig. 7a to b), creating a more enclosed pocket in ToxT (Fig. 7c) than in Rns, which has an opening in the side of the pocket (Fig. 7d). Both pockets are predominantly hydrophobic except for the charged groups involved in binding the carboxyl portion of the fatty acid (Fig. 7c,d). Palmitoleate occupies a larger portion of the binding pocket in ToxT than the decanoate in Rns (Fig. 7c,d), suggesting Rns is able to bind ligands larger than decanoate. While fatty acids regulate both proteins, the details of the binding are different, which is likely to be a theme among fatty acid regulated AraC’s.

## Discussion

As part of ongoing efforts to determine whether other AraC proteins are regulated by fatty acids in a similar fashion as ToxT, we solved the structure of Rns using X-ray crystallography in two conformations: open, distinguished by having a groove between the N and C-terminal domains for ligand binding; and closed, where the two domains have come closer together with the pocket enclosing the ligand. The Rns structures were dimeric, with an interface similar to structures of other AraC proteins^30,31^. The DNA binding domains of the monomers are pointing ~ 180° away from each other, suggesting Rns binds DNA loops in a manner similar to AraC^53^. Such an orientation is also consistent with the observation that most Rns regulated promoters have proximal and distal sites separated by about forty base pairs, although for some promoters this separation is less and for others it is more^16,19,23,42,45,56^. For example, CS1 pili expression is mediated by two sites, one proximal to the -35 RNAP binding site, and one distal, around − 106, to the transcription start site^23^. The sites are asymmetric and contribute additively to the expression of the CS1 pili. The *rns* promoter has two binding sites, one at the − 227 position and the other downstream of the transcription start site^56^, which are widely spaced and act synergistically, with both required for activating transcription^56^. These findings, coupled with the observations from our structures, indicate Rns likely binds to looped DNA. Work is ongoing to investigate the relevance of this interface and to clarify if Rns binds looped DNA.

The ligand binding pocket of Rns was occupied by the 10-carbon fatty acid, decanoic acid. DSF studies showed decanoic acid specifically stabilizes Rns, and that it inhibits Rns activated expression of CexE as well as the CS3 and CFA/I major pilins in ETEC strains. Decanoic acid also inhibits Rns activity at the *cexE*, CFA/I, and CS3 promoters, where Rns activates transcription, and at the *nlpA* promoter, where Rns represses transcription^16,42^. Therefore, we have identified the first small molecule that inhibits Rns mediated expression of ETEC virulence factors. Based on homology, we expect these results to be applicable to CsvR, which is found in other ETEC strains, and AggR from enteroaggregative *E. coli*^17,57^.

The finding that decanoic acid inhibits expression of ETEC colonization factors raises several questions including, what is the source of the decanoic acid, what are its physiological concentrations, and where in the intestine would ETEC be exposed to the molecule? One source of decanoic acid is food, including milk products, coconut oil, palm kernel oil, and some vegetable oils^58–61^. Decanoic acid, like all medium chain fatty acids, is rapidly absorbed by the small intestine, resulting in a decreasing concentration gradient of dietary sourced decanoate along the intestinal tract^62,63^. In addition to diet, decanoic acid derivatives may be synthesized by the microbiota for signaling purposes. For example, it is known *Psuedomonas aeruginosa* produces cis-2-decenoic acid to signal biofilm dispersal^64,65^, and it is unlikely to be only bacteria to produce such molecules. Furthermore, while we identified decanoic acid as a Rns ligand, the structure of the Rns binding pocket suggests a number of relevant natural products could be capable of binding to and regulating the protein. While the specific structural mechanism by which Rns is regulated by decanoic acid is not clear, it likely involves influencing the stability of the Rns dimer, either directly or by a dynamic allosteric mechanism as demonstrated for *V. cholerae* ToxT^66^, and future studies will be directed at clarifying this mechanism.

This study demonstrates the first time that a small molecule binds to Rns and inhibits virulence gene expression in ETEC, and provides a foundation for decanoic acid and its analogs to be pursued as anti-virulence lead compounds for limiting the morbidity and mortality caused by ETEC.

## Supplementary Information

Supplementary Information.

## Data Availability

The structures of Rns have been deposited into the PDB: SeMet-Rns; 6XIV, and Rns; 6XIU.

## References

[1] Troeger C (2017). Estimates of global, regional, and national morbidity, mortality, and aetiologies of diarrhoeal diseases: A systematic analysis for the Global Burden of Disease Study 2015. Lancet Infect. Dis..

[2] World Health Organization (1999). New frontiers in the development of vaccines against enterotoxinogenic (ETEC) and enterohaemorrhagic (EHEC) *E. coli* infections. Part I. Wkly. Epidemiol. Rec..

[3] Khalil IA (2018). Morbidity and mortality due to shigella and enterotoxigenic *Escherichia coli* diarrhoea: The Global Burden of Disease Study 1990–2016. Lancet Infect. Dis..

[4] Porter CK, Olson S, Hall A, Riddle MS (2017). Travelers’ diarrhea: An update on the incidence, etiology, and risk in military deployments and similar travel populations. Mil. Med..

[5] Jiang ZD, DuPont HL (2017). Etiology of travellers' diarrhea. J. Travel Med..

[6] Satterwhite TK, Evans DG, DuPont HL, Evans DJ (1978). Role of *Escherichia coli* colonisation factor antigen in acute diarrhoea. The Lancet.

[7] Evans DG, Satterwhite TK, Evans DJ, DuPont HL (1978). Differences in serological responses and excretion patterns of volunteers challenged with enterotoxigenic *Escherichia coli* with and without the colonization factor antigen. Infect. Immun..

[8] Turner SM, Scott-Tucker A, Cooper LM, Henderson IR (2006). Weapons of mass destruction: Virulence factors of the global killer enterotoxigenic *Escherichia coli*. FEMS Microbiol. Lett..

[9] Fleckenstein JM, Kuhlmann FM (2019). Enterotoxigenic *Escherichia coli* infections. Curr. Infect. Dis. Rep..

[10] Boedeker EC (2005). Vaccines for enterotoxigenic *Escherichia coli*: Current status. Curr. Opin. Gastroenterol..

[11] Vidal RM (2019). Colonization factors among enterotoxigenic *Escherichia coli* isolates from children with moderate-to-severe diarrhea and from matched controls in the Global Enteric Multicenter Study (GEMS). PLoS Negl. Trop. Dis..

[12] Qadri F (2020). Safety and immunogenicity of the oral, inactivated, enterotoxigenic *Escherichia coli* vaccine ETVAX in Bangladeshi children and infants: A double-blind, randomised, placebo-controlled phase 1/2 trial. Lancet Infect. Dis..

[13] Shakoor S, Platts-Mills JA, Hasan R (2019). Antibiotic-resistant enteric infections. Infect. Dis. Clin. N. Am..

[14] Caron J, Scott JR (1990). A *rns*-like regulatory gene for colonization factor antigen I (CFA/I) that controls expression of CFA/I pilin. Infect. Immun..

[15] Caron J, Coffield LM, Scott JR (1989). A plasmid-encoded regulatory gene, *rns*, required for expression of the CS1 and CS2 adhesins of enterotoxigenic *Escherichia coli*. Proc. Natl. Acad. Sci..

[16] Bodero MD, Harden EA, Munson GP (2008). Transcriptional regulation of subclass 5b fimbriae. BMC Microbiol..

[17] de Haan LA, Willshaw GA, van der Zeijst BA, Gaastra W (1991). The nucleotide sequence of a regulatory gene present on a plasmid in an enterotoxigenic *Escherichia coli* strain of serotype O167:H5. FEMS Microbiol. Lett..

[18] Munson GP (2013). Virulence regulons of enterotoxigenic *Escherichia coli*. Immunol. Res..

[19] Bodero MDR, Munson GP (2016). The virulence regulator Rns activates the expression of CS14 pili. Genes (Basel).

[20] Willshaw GA, McConnell MM, Smith HR, Rowe B (1990). Structural and regulatory genes for coli surface associated antigen 4 (CS4) are encoded by separate plasmids in enterotoxigenic *Escherichia coli* strains of serotype 0.25.H42. FEMS Microbiol. Lett..

[21] Gallegos M-T, Schleif R, Bairoch A, Hofmann K, Ramos JL (1997). Arac/XylS family of transcriptional regulators. Microbiol. Mol. Biol. Rev..

[22] Grewal HM, Gaastra W, Svennerholm A-M, Röli J (1993). Induction of colonization factor antigen I (CFA/I) and coli surface antigen 4 (CS4) of enterotoxigenic *Escherichia coli*: Relevance for vaccine production. Vaccine.

[23] Munson GP, Scott JR (1999). Binding site recognition by Rns, a virulence regulator in the AraC family. J. Bacteriol..

[24] Higgins DE, Nazareno E, DiRita VJ (1992). The virulence gene activator ToxT from *Vibrio cholerae* is a member of the AraC family of transcriptional activators. J. Bacteriol..

[25] Mahon V, Smyth CJ, Smith SGJ (2010). Mutagenesis of the Rns regulator of enterotoxigenic *Escherichia coli* reveals roles for a linker sequence and two helix-turn-helix motifs. Microbiology (Reading).

[26] DiRita VJ, Parsot C, Jander G, Mekalanos JJ (1991). Regulatory cascade controls virulence in *Vibrio cholerae*. Proc. Natl. Acad. Sci..

[27] Lowden MJ (2010). Structure of *Vibrio cholerae* ToxT reveals a mechanism for fatty acid regulation of virulence genes. Proc. Natl. Acad. Sci. U.S.A..

[28] Chatterjee A, Dutta PK, Chowdhury R (2007). Effect of fatty acids and cholesterol present in bile on expression of virulence factors and motility of *Vibrio cholerae*. Infect. Immun..

[29] LaRonde-LeBlanc N, Wolberger C (2000). Characterization of the oligomeric states of wild type and mutant AraC. Biochemistry.

[30] Soisson SM, MacDougall-Shackleton B, Schleif R, Wolberger C (1997). Structural basis for ligand-regulated oligomerization of AraC. Science.

[31] Shrestha M, Xiao Y, Robinson H, Schubot FD (2015). Structural analysis of the regulatory domain of ExsA, a key transcriptional regulator of the type three secretion system in *Pseudomonas aeruginosa*. PLoS ONE.

[32] Ibarra JA, Perez-Rueda E, Segovia L, Puente JL (2008). The DNA-binding domain as a functional indicator: The case of the AraC/XylS family of transcription factors. Genetica.

[33] Studier FW (2005). Protein production by auto-induction in high-density shaking cultures. Protein Expr. Purif..

[34] Kabsch W (2010). XDS. Acta Crystallogr. D Biol. Crystallogr..

[35] Adams PD (2010). PHENIX: A comprehensive python-based system for macromolecular structure solution. Acta Crystallogr. D Biol. Crystallogr..

[36] Emsley P, Cowtan K (2004). Coot: Model-building tools for molecular graphics. Acta Crystallogr. D Biol. Crystallogr..

[37] Pettersen EF (2004). UCSF chimera—A visualization system for exploratory research and analysis. J. Comp. Biol..

[38] Goddard TD (2017). UCSF ChimeraX: Meeting modern challenges in visualization and analysis. Protein Sci..

[39] McCoy AJ (2007). Phaser crystallographic software. J. Appl. Cryst..

[40] Niesen FH, Berglund H, Vedadi M (2007). The use of differential scanning fluorimetry to detect ligand interactions that promote protein stability. Nat. Protoc..

[41] Midgett CR (2017). Bile salts and alkaline pH reciprocally modulate the interaction between the periplasmic domains of *Vibrio cholerae* ToxR and ToxS. Mol. Microbiol..

[42] Bodero MD, Pilonieta MC, Munson GP (2007). Repression of the inner membrane lipoprotein NlpA by Rns in enterotoxigenic *Escherichia coli*. J. Bacteriol..

[43] Haldimann A, Wanner BL (2001). Conditional-replication, integration, excision, and retrieval plasmid-host systems for gene structure-function studies of bacteria. J. Bacteriol..

[44] Basturea GN, Bodero MD, Moreno ME, Munson GP (2008). Residues near the amino terminus of Rns are essential for positive autoregulation and DNA binding. J. Bacteriol..

[45] Pilonieta MC, Bodero MD, Munson GP (2007). CfaD-dependent expression of a novel extracytoplasmic protein from enterotoxigenic *Escherichia coli*. J. Bacteriol..

[46] Casadaban MJ (1976). Transposition and fusion of the *lac* genes to selected promoters in *Escherichia coli* using bacteriophage lambda and Mu. J. Mol. Biol..

[47] Datsenko KA, Wanner BL (2000). One-step inactivation of chromosomal genes in *Escherichia coli* K-12 using PCR products. Proc. Natl. Acad. Sci..

[48] Rivas ZP (2020). CexE Is a coat protein and virulence factor of diarrheagenic pathogens. Front. Microbiol..

[49] Miller JH (1972). Experiments in Molecular Genetics.

[50] Skerman FJ, Formal SB, Falkow S (1972). Plasmid-associated enterotoxin production in a strain of *Escherichia coli* isolated from humans. Infect. Immun..

[51] Adlerberth I (1995). Adhesins of *Escherichia coli* associated with extra-intestinal pathogenicity confer binding to colonic epithelial cells. Microb. Pathog..

[52] Frank DW, Iglewski BH (1991). Cloning and sequence analysis of a trans-regulatory locus required for exoenzyme S synthesis in *Pseudomonas aeruginosa*. J. Bacteriol..

[53] Lobell RB, Schleif RF (1990). DNA looping and unlooping by AraC protein. Science.

[54] Belmont-Monroy L (2020). Characterization of a novel AraC/XylS-regulated family of N-acyltransferases in pathogens of the order Enterobacterales. PLoS Pathog..

[55] Icke C (2021). Glycine acylation and trafficking of a new class of bacterial lipoprotein by a composite secretion system. Elife.

[56] Munson GP, Scott JR (2000). Rns, a virulence regulator within the AraC family, requires binding sites upstream and downstream of its own promoter to function as an activator. Mol. Microbiol..

[57] Nataro JP, Yikang D, Yingkang D, Walker K (1994). AggR, a transcriptional activator of aggregative adherence fimbria I expression in enteroaggregative *Escherichia coli*. J. Bacteriol..

[58] Deen A (2021). Chemical composition and health benefits of coconut oil: an overview. J. Sci. Food Agric..

[59] Jensen RG (2002). The composition of bovine milk lipids: January 1995 to December 2000. J. Dairy Sci..

[60] Nainggolan M, Sinaga AGS (2021). Characteristics of fatty acid composition and minor constituents of red palm olein and palm kernel oil combination. J. Adv. Pharm. Technol. Res..

[61] Alves AQ (2019). The fatty acid composition of vegetable oils and their potential use in wound care. Adv. Skin Wound Care.

[62] Caspary WF (1992). Physiology and pathophysiology of intestinal absorption. Am. J. Clin. Nutr..

[63] Banwell JG, Gorbach SL, Pierce NF, Mitra R, Mondal A (1971). Acute undifferentiated human diarrhea in the tropics: II. Alterations in intestinal fluid and electrolyte movements. J. Clin. Investig..

[64] Davies DG, Marques CNH (2009). A fatty acid messenger is responsible for inducing dispersion in microbial biofilms. J. Bacteriol..

[65] Marques CNH, Davies DG, Sauer K (2015). Control of biofilms with the fatty acid signaling molecule cis-2-decenoic acid. Pharmaceuticals (Basel).

[66] Cruite JT (2019). Structural basis for virulence regulation in *Vibrio cholerae* by unsaturated fatty acid components of bile. Commun. Biol..

