# Supplementary material for: Structure of the master regulator Rns reveals an inhibitor of enterotoxigenic Escherichia coli virulence regulons
Source: Sci Rep. 2021 Aug 2;11:15663. doi: 10.1038/s41598-021-95123-2 (PMC8329261; doi:10.1038/s41598-021-95123-2)

Table S1: Refinement Statistics		
	**SeMet-Rns**	**Rns**
Wavelength		
Resolution range	29.29 - 2.8 (2.9 - 2.8)	27.39 - 3.0 (3.107 - 3.0)
Space group	P 21	P 21 21 21
Unit cell	72.51 49.97 102.92 90 106.11 90	48.24 95.15 133.09 90 90 90
Total reflections	228834 (24784)	81535 (8041)
Unique reflections	17538 (1743)	12798 (1219)
Multiplicity	13.0 (14.2)	6.4 (6.6)
Completeness (%)	94.93 (86.50)	99.16 (97.50)
Mean I/sigma(I)	8.67 (0.74)	7.29 (0.88)
Wilson B-factor	89.24	93.97
R-merge	0.1984 (3.508)	0.1518 (1.994)
R-meas	0.2069 (3.637)	0.1654 (2.162)
R-pim	0.0579 (0.956)	0.06498 (0.8261)
CC1/2	0.995 (0.29)	0.997 (0.549)
CC*	0.999 (0.671)	0.999 (0.842)
Reflections used in refinement	16917 (1532)	12745 (1211)
Reflections used for R-free	1679 (143)	1277 (120)
R-work	0.2422 (0.3928)	0.2571 (0.3840)
R-free	0.2999 (0.4694)	0.3007 (0.4249)
CC(work)	0.948 (0.589)	0.955 (0.646)
CC(free)	0.911 (0.461)	0.915 (0.423)
Number of non-hydrogen atoms	4222	4068
macromolecules	4189	4044
ligands	33	24
Protein residues	513	494
RMS(bonds)	0.008	0.012
RMS(angles)	1.17	1.36
Ramachandran favored (%)	93.89	92.39
Ramachandran allowed (%)	5.92	7.2
Ramachandran outliers (%)	0.2	0.41
Rotamer outliers (%)	4.99	6.9
Clashscore	7.4	19.06
Average B-factor	93.97	83.91
macromolecules	94.01	84.04
ligands	89.13	61.91

Table S2: Plasmids used in this study			
**Name**	**Description**	**Marker**	**Reference**
pTags2	Cloning vector	*bla*	Addgene
pGPMRns	Rns expressed from *lacp*	*bla*	[1]
pGPMRns-Myc	Rns-Myc expressed from *lacp*	*bla*	This study
pHKLac1	Integration plasmid	*aadA*	[2]
pCS3Lac1	Integration plasmid [*cs3p*(-121 to +352 relative to ORF)*::lacZ*]	*aadA*	This study
pCFAILac1	Integration plasmid [*cfaAp*(-486 to +343 relative to ORF)::*lacZ*]	*aadA*	[3]
pCexELac1	Integration plasmid [*cexEp*(-549 to +264 relative to ORF)::*lacZ*]	*aadA*	[4]
pNlpALac1	Integration plasmid [*nlpAp*(-391 to +58 relative to ORF*)::lacZ*].	*aadA*	[1]
pAH162	PCR template for tetracycline cassette	*tet*	[5]

Table S3: Oligonucleotides used in this study

Name	Sequence
38	AAAGGATCCCGAAGCCGGTACCC
40	AAAGAATTCCGCCTCAAAATATACTC
394	GCTGGATCCCGAGCGGCGTATAAAA
395	GCTGGATCCCGAGCGGCGTATAAAA
401	GATGGATCCCATGAAAATACGCACGCG
402	GTGGAATTCCTCGCACACAGAGGG
415	GCAGGATCCCACGCTTCATCTGATAATGACG
416	GCAGAATTCCGGCCAGCAATAATG
1150	**GCACTGGAAATTCCAATCATATTTGATATCTGAGATATCTGGTATGAATTTTCAAGTAGT**TGCTGATCTTCAGATCCTC
1242	**CAATAAGTTGGAGTCATTACCAGTGCGTTTAATATAATATTCATTAGCGGTATTATGATG**CTACCATCGGGGGCC
1414	CATATGTATATCTCCTTCTTTGGCC
1419	GAACAGAAACTGATTAGCGAAG
1519	gccaaagaaggagatatacatATGGACTTTAAATACACTGAAG
1522	cgctaatcagtttctgttcTCCACCTTTAAAATAAGTGAAAAATTG

**Underlining indicates primer-template mismatches that add sites for restriction endonucleases. Bold indicates λ Red targeting region. Lowercase denotes target sequences for HiFi assembly.*

Table S4: Strains used in this study		
**Strain**	**Characteristics**	**Reference**
MC4100	*E. coli* K-12 *F- araD139 Δ(argF-lac)U169 rpsL150* (StrR*) relA1 flhD5301 deoC1 ptsF25 rbsR*	[6]
GPM1061	MC4100 *attB*$_{HK022}$::pCFAILac1	[3]
GPM1070	MC4100 *attB*$_{HK022}$::pCexELac1	[4]
GPM1072	MC4100 *attB*$_{HK022}$::pCS3Lac1	This study
GPM1080	MC4100 *attB*$_{HK022}$::pNlpALac1	[1]
GPM1710	BW25113 *attB*$_{HK022}$::pTibDBLac1	[7]
H10407	ETEC O78:H11 CFA/I+ ST+ LT+ CexEα+	[8]
1392/75-2a	ETEC O6:H16 CS1+ CS3+ ST+ LT+ CexEκ+ CexEε+	[9]
GPM1236	H10407 *cfaD::kan*	[10]
GPM3002	1392/75-2a *rns::tet*	This study

Table References:

1. Bodero, M. D., Pilonieta, M. C. & Munson, G. P. Repression of the inner membrane lipoprotein NlpA by Rns in enterotoxigenic *Escherichia coli*. *Journal of Bacteriology* **189**, 1627–1632 (2007).
2. Haldimann, A. & Wanner, B. L. Conditional-replication, integration, excision, and retrieval plasmid-host systems for gene structure-function studies of bacteria. *Journal of Bacteriology* **183**, 6384–6393 (2001).
3. Basturea, G. N., Bodero, M. D., Moreno, M. E. & Munson, G. P. Residues near the amino terminus of Rns are essential for positive autoregulation and DNA binding. *Journal of Bacteriology* **190**, 2279–2285 (2008).
4. Pilonieta, M. C., Bodero, M. D. & Munson, G. P. CfaD-dependent expression of a novel extracytoplasmic protein from enterotoxigenic *Escherichia coli*. *Journal of Bacteriology* **189**, 5060–5067 (2007).
5. Datsenko, K. A. & Wanner, B. L. One-step inactivation of chromosomal genes in *Escherichia coli* K-12 using PCR products. *Proceedings of the National Academy of Sciences* **97**, 6640–6645 (2000).

6. Casadaban, M. J. Transposition and fusion of the *lac* genes to selected promoters in *Escherichia coli* using bacteriophage lambda and Mu. *J Mol Biol* **104**, 541–555 (1976).
7. Espert, S. M., Elsinghorst, E. A. & Munson, G. P. The tib adherence locus of enterotoxigenic *Escherichia coli* is regulated by cyclic AMP receptor protein. *Journal of Bacteriology* **193**, 1369–1376 (2011).
8. Skerman, F. J., Formal, S. B. & Falkow, S. Plasmid-associated enterotoxin production in a strain of Escherichia coli isolated from humans. *Infection and Immunity* **5**, 622–624 (1972).
9. Adlerberth, I. *et al.* Adhesins of *Escherichia coli* associated with extra-intestinal pathogenicity confer binding to colonic epithelial cells. *Microb. Pathog.* **18**, 373–385 (1995).
10. Rivas, Z. P. *et al.* CexE Is a coat protein and virulence factor of diarrheagenic pathogens. *Front. Microbiol.* **11**, 1374 (2020).

Fig S1: *Full western blots used in figure 4.* One blot for each strain was probed sequentially first for CexE then for loading control DnaK. The bands corresponding to CexE and DnaK are labeled. On the far left of each blot is an antibody control lane *cexE::kan* for both strains.

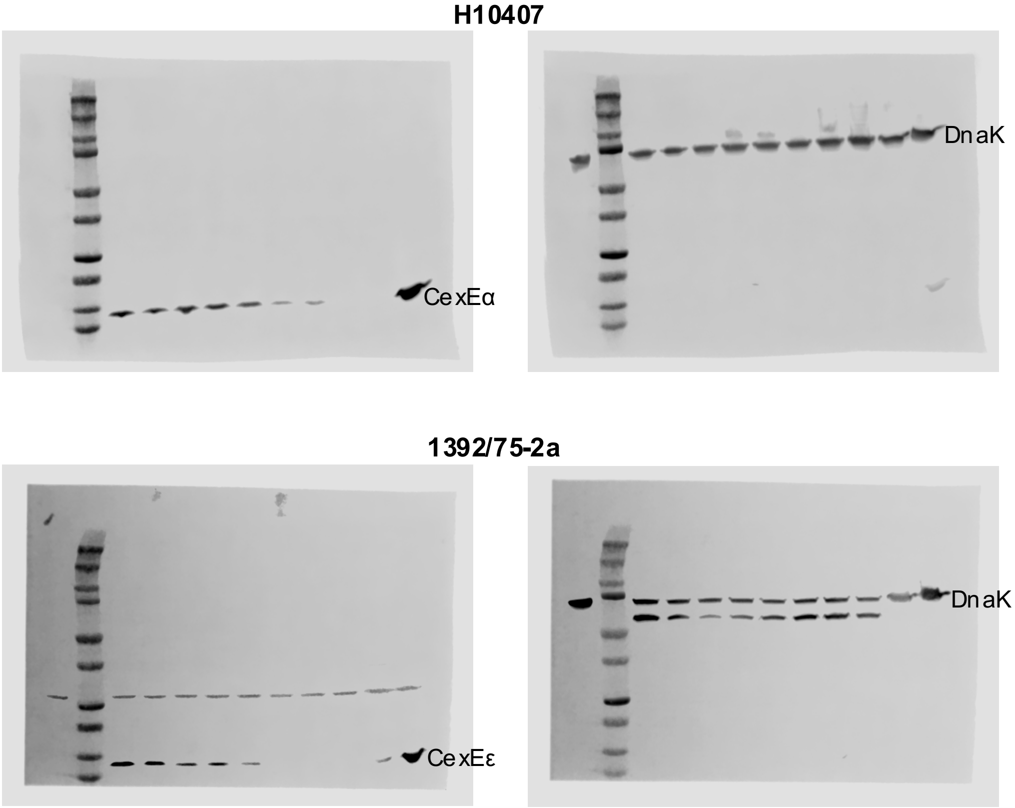

Fig S2: *The effect of decanoic acid on bacterial growth.* Strains H10407, 1392/75-2a, and MC4100 were grown in LB with 0, 1.25, and 5 mM decanoic acid (w/v, 0.4% final DMSO) in triplicate. Data is given as the mean OD600 ± SD, n=3.

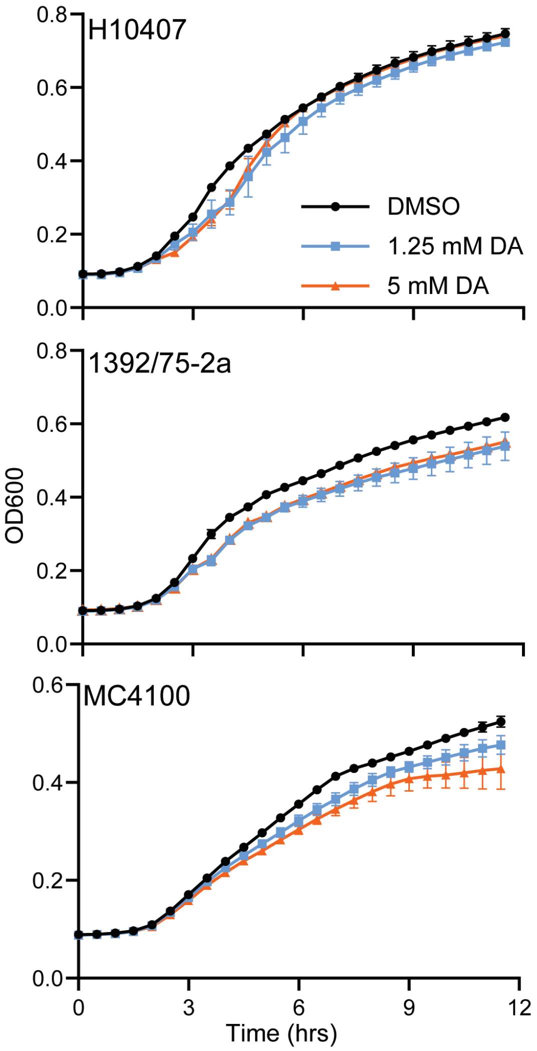

Fig S3: *Decanoic acid has a limited impact on the Rns-independent promoter tibDB*. Strain GPM1710 was grown to stationary phase aerobically at 37 °C overnight then tested for β-galactosidase expression. Although off target effects were observed, the magnitude was less compared to Rns-dependent promoters.

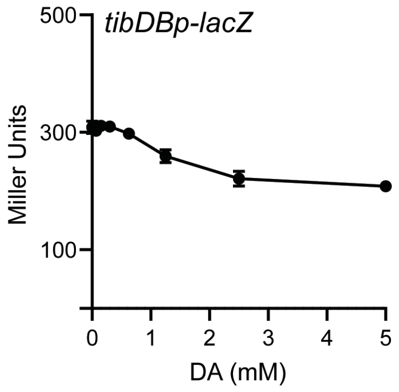

Supplement: Supplementary file 1 — Supplementary Information. [file 41598_2021_95123_MOESM1_ESM.pdf]
